# Characterization of *Plasmodium vivax* Early Transcribed Membrane Protein 11.2 and Exported Protein 1

**DOI:** 10.1371/journal.pone.0127500

**Published:** 2015-05-26

**Authors:** Yang Cheng, Feng Lu, Seong-Kyun Lee, Deok-Hoon Kong, Kwon-Soo Ha, Bo Wang, Jetsumon Sattabongkot, Takafumi Tsuboi, Eun-Taek Han

**Affiliations:** 1 Department of Medical Environmental Biology and Tropical Medicine, School of Medicine, Kangwon National University, Chuncheon, Gangwon-do, Republic of Korea; 2 Laboratory of Malaria and Vector Research, National Institute of Allergy and Infectious Diseases (NIAID), National Institutes of Health (NIH), Rockville, Maryland, United States of America; 3 Key Laboratory of Parasitic Disease Control and Prevention (Ministry of Health), Jiangsu Institute of Parasitic Diseases, Wuxi, Jiangsu Province, People’s Republic of China; 4 Jiangsu Provincial Key Laboratory of Parasite Molecular Biology, Jiangsu Institute of Parasitic Diseases, Wuxi, Jiangsu Province, People’s Republic of China; 5 Department of Molecular and Cellular Biochemistry, School of Medicine, Kangwon National University, Chuncheon, Gangwon-do, Republic of Korea; 6 Department of Clinical Laboratory, The First Affiliated Hospital of Anhui Medical University, Hefei, Anhui, People’s Republic of China; 7 Mahidol Vivax Research Unit, Faculty of Tropical Medicine, Mahidol University, Bangkok, Thailand; 8 Division of Malaria Research, Proteo-Science Center, Ehime University, Matsuyama, Ehime, Japan; Agency for Science, Technology and Research—Singapore Immunology Network, SINGAPORE

## Abstract

In *Plasmodium*, the membrane of intracellular parasites is initially formed during invasion as an invagination of the red blood cell surface, which forms a barrier between the parasite and infected red blood cells in asexual blood stage parasites. The membrane proteins of intracellular parasites of *Plasmodium* species have been identified such as early-transcribed membrane proteins (ETRAMPs) and exported proteins (EXPs). However, there is little or no information regarding the intracellular parasite membrane in *Plasmodium vivax*. In the present study, recombinant PvETRAMP11.2 (PVX_003565) and PvEXP1 (PVX_091700) were expressed and evaluated antigenicity tests using sera from *P*. *vivax*-infected patients. A large proportion of infected individuals presented with IgG antibody responses against PvETRAMP11.2 (76.8%) and PvEXP1 (69.6%). Both of the recombinant proteins elicited high antibody titers capable of recognizing parasites of vivax malaria patients. PvETRAMP11.2 partially co-localized with PvEXP1 on the intracellular membranes of immature schizont. Moreover, they were also detected at the apical organelles of newly formed merozoites of mature schizont. We first proposed that these proteins might be synthesized in the preceding schizont stage, localized on the parasite membranes and apical organelles of infected erythrocytes, and induced high IgG antibody responses in patients.

## Introduction

Malaria is caused by blood infection with *Plasmodium* parasites, which are transmitted by *Anopheles* mosquitoes. During parasite invasion and development within host cells, parasitophorous vacuoles (PVs) are formed for parasite intracellular survival, growth and development. The PV membrane (PVM) plays a critical role in nutrient acquisition, host cell remodeling, waste disposal, environmental sensing, and protection from innate defense [1].

PVMs originate from infected RBC (iRBC) plasma membranes and include iRBC membrane lipid raft proteins and parasite-coded proteins [2]. A number of blood-stage parasite-coded proteins are involved in *Plasmodium falciparum* PVM formation, including early-transcribed membrane proteins (ETRAMPs) [3, 4], the *Plasmodium* translocon for exported proteins (PTEX) [5, 6], exported protein 1 (EXP1) [7, 8] and the high molecular mass rhoptry protein (RhopH)/ cytoadherence-linked asexual gene (CLAG) complex [9, 10]. To date, the precise role of ETRAMPs is unclear. However, they might constitute the prominent protein of the PVM of blood-stage parasites [3, 4]. It was shown that RhopH2 was delivered to PVM after invasion [11], and CLAGs were also involved in nutrient uptake at both the host cell surface and the PVM would be conceivable [12]. EXP1 is inserted into the PVM with its C-terminus facing the host cell cytoplasm [13], where it forms oligomers in *P*. *falciparum* parasites [14]. However, it is not clearly understood those of parasite membrane formation in parasite-infected RBCs of *Plasmodium vivax* because of limitation of continuous in vitro culture system.

In our previous studies, we screened immunogenic candidates from 232 blood-stage proteins of *P*. *vivax* parasites using sera samples from vivax patients that were based on the sequences of the signal peptide (SP), transmembrane (TM) domain or orthologs of well-known *P*. *falciparum* proteins [15, 16]. Among them, PvETRAMP11.2 (PVX_003565) and PvEXP1 (PVX_091700) were identified as immunogenic candidates, and their orthologs were proven to be PVM-associated proteins [17]. Herein, we first report that PvETRAMP11.2 partially colocalizes with PvEXP1 in the intracellular parasite membrane of immature schizont stage, and both of them could be detected in apical organelles in mature schizont-stage parasites. Both of recombinant proteins elicited high antibody responses from vivax malaria patients. In addition, PvETRAMP11.2 possibly interacts with PvEXP1 in *P*. *vivax* parasites. Collectively, our results confirmed that PvETRAMP11.2, and PvEXP1 are immunogenic in vivax malaria patients during natural infection and may involve in constitutes of intracellular parasite membrane during growth and development of *P*. *vivax* parasites.

## Methods

### Ethics

This study was approved by the Institutional Review Board at Kangwon National University Hospital. A signed informed consent was obtained from each subject enrolled in this study (Approval No. 10-041-07). All animal experimental protocols were approved by the Institutional Animal Care and Use Committee of Kangwon National University, and the experiments were conducted according to the Ethical Guidelines for Animal Experiments of Kangwon National University (KIACUC-13-0001).

### Human sera samples

Sera samples were collected from 56 patients (mean age, 24 years; range 18–42 years) with the symptoms and positive signs by microscopy of vivax malaria (mean parasitemia, 0.117%; range 0.012–0.37%). Samples were obtained from local health centers and clinics in Gyeonggi and Gangwon provinces in endemic areas of the Republic of Korea (ROK). Forty sera samples of healthy individuals, negative by microscopy, were collected in nonendemic areas of ROK. Sera samples were separated from whole blood and used for this study. This study was approved by the Institutional Review Board at Kangwon National University Hospital. Genomic DNA from the parasite was prepared from 200 μl of whole blood from a *P*. *vivax* patient in ROK using a QIAamp DNA Blood Mini Kit (Qiagen, Hilden, Germany), which provided 200 μl aliquots of template DNA.

### Enrichment of parasite-infected erythrocytes for parasite antigens

*P*. *vivax-*infected blood samples were collected from patients, and parasite-infected erythrocytes were purified as in a previous report [18]. Infected patient samples were used to remove of white blood cells with Plasmodipur filter (Euro-Diagnostica, Arnhem, The Netherlands). They were then resuspended in RPMI1640 medium (Invitrogen, Carlsbad, CA, USA) to make a 10% hematocrit suspension of erythrocytes. Thereafter, schizont-rich infected erythrocytes were enriched by 60% Percoll gradient centrifugation and used as parasite antigens for Western blot and immunofluorescent analyses.

### Expression and purification of recombinant proteins

**Fig 1:**
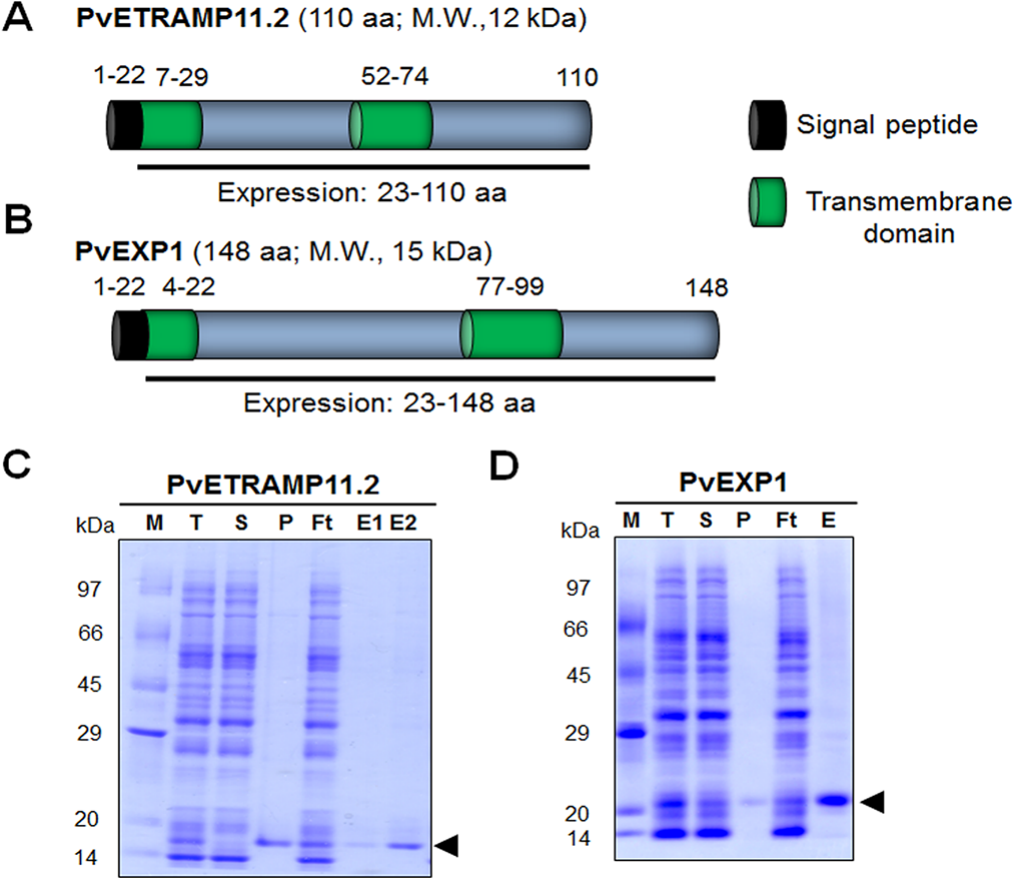
Schematic structures, expression and purification of PvETRAMP11.2 and PvEXP1. *A*. The PvETRAMP11.2 protein comprises 110 amino acids (AAs), with a calculated molecular mass of 12 kDa. Indicated are the signal peptide (AA positions 1–22) and two transmembrane domains (AAs 7–29 and 52–74, respectively). Truncated PvETRAMP11.2 (AAs 23–110) was constructed for expression. *B*. The PvEXP1 protein comprises 148 AAs, with a calculated molecular mass of 15 kDa. Indicated are the signal peptide (AAs 1–22) and two transmembrane domains (AAs 4–22 and 77–99, respectively). Truncated PvEXP1 (AAs 23–148) was constructed for expression. Purified recombinant PvETRAMP11.2 (*C*) and PvEXP1 (*D*) were resolved by 12% SDS-PAGE. M, protein marker; T, total translation mix; S, supernatant; P, pellet; Ft: flow through; E1, elution fraction 1; E2, elution fraction 2; kDa, kilo Dalton; arrow heads, purified proteins.

Recombinant PvETRAMP11.2 (PVX_003565) and PvEXP1 (PVX_091700) proteins were designed based on the *P*. *vivax* Sal-1 strain sequence on the PlasmoDB website (www.plasmodb.org) and amplified from the genomic DNA of *P*. *vivax* isolates from ROK. Here, we expressed and purified the PvETRAMP11.2 (amino acids [AAs], 23–74] and PvEXP1 (AAs, 23–148) proteins (Fig 1) lacking the SP using wheat germ cell-free (WGCF) expression [19]. Briefly, *pvetramp11*.*2* and *pvexp1* DNA fragments were amplified using the following primers: 5′-GGGCGGATATCTCGAGTTCTACAATAATGTTGTAGCAGGAAAG-3′ and 5′-GCGGTACCCGGGATCCTTATTGGATGTTGCTGCCTTT-3′, 5′-GGGCGGATATCTCGAGAATGTAAACGGGTTAGGTGCTG-3′ and 5′-GCGGTACCCGGGATCCTCATGACGTTGATTCGGTG-3′. The underlined primer sequence indicates it is homologous to the vector sequence. They were then cloned into the pEU-E01-His-TEV-MCS vector (CellFree Sciences, Matsuyama, Japan) by In-Fusion Cloning (Clontech, Palo Alto, CA, USA) and the cloned inserts were sequenced using an ABI 3700 Genetic Analyzer (Applied Biosystems, Inc., Foster City, CA, USA) by Genotech (Daejon, Korea). These proteins were expressed using a WGCF system and purified using a Ni-Sepharose column as described [20].

### Animal immunization

Female BALB/c mice (Daehan Biolink Co., Eumsung, Korea) were used at 6–8 weeks of age. Groups of three mice were injected intraperitoneally with about 20 μg of each purified recombinant protein with Freund’s complete adjuvant (Sigma-Aldrich, St. Louis, MO, USA). Booster injections were given 3 and 6 weeks later using the same amount of antigen with Freund’s incomplete adjuvant (Sigma-Aldrich). Mouse blood samples were taken 2 weeks after the final booster. In addition, two Japanese white rabbits were immunized subcutaneously with 250 μg of purified recombinant proteins with Freund's complete adjuvant, followed by 250 μg proteins with Freund’s incomplete adjuvant. All immunizations were conducted three times at 3-week intervals. The antisera were collected 14 days after the last immunization.

### SDS-PAGE and Western blot analysis

PvETRAMP11.2 and PvEXP1 recombinant proteins (1 μg each) or *P*. *vivax* parasite extracts (10 μg) were separated using 12% SDS-PAGE after denaturation with the reducing agent β-mercaptoethanol in sample buffer and then stained with Coomassie brilliant blue. For Western blot analysis, recombinant proteins were transferred electrophoretically to PVDF membranes (Millipore, Bedford, MA, USA), and incubated with blocking buffer (5% nonfat milk in PBS containing 0.2% Tween 20, PBS-T) for 1 h at 37°C. After blocking, penta anti-His antibody (1:1000), mouse immune sera (1:200), rabbit immune serum (1:1000) or vivax-infected patient serum (1:500) diluted into PBS-T and secondary IRDye goat anti-mouse, IRDye goat anti-rabbit antibodies, or IRDye goat anti-human antibodies (LI-COR Biosciences, Lincoln, NE, USA), respectively, were used to detect His-tagged recombinant proteins and specific immune sera quality. Data were scanned by an Odyssey infrared imaging system (LI-COR Biosciences) and analyzed by Odyssey software (LI-COR Biosciences).

### Protein arrays

Amine-coated slides were prepared as described previously [15]. To develop protein arrays, sera samples from 56 cases of vivax malaria and 40 unexposed individuals were used for humoral immune response analyses using well-type amine arrays. A series of double dilutions was developed to optimize the coating concentration (0.1–200 μg/ml) of PvETRAMP11.2, and PvEXP1. The purified recombinant PvETRAMP11.2 and PvEXP1 proteins were spotted onto the duplicate wells of the arrays in PBS at 50 and 100 μg/ml, respectively, and incubated for 1 h at 37°C. Each well was blocked with 1.0 μl of blocking buffer (5% BSA in PBS with 0.1% Tween 20, PBS-T) and incubated for 1 h at 37°C. The chips were pre-absorbed against wheat germ lysate (1:100 dilution) to block anti-wheat germ antibodies and then probed with human malaria patients or healthy individuals (1:200 dilution). Alexa Fluor 546 goat anti-human IgG (10 μg/ml, Invitrogen) in PBS-T was used to detect antibodies, which was quantified as described previously, and the antibodies were scanned in a fluorescence scanner (ScanArray Express; PerkinElmer, Boston, MA, USA) [15]. The cutoff value was equal to the mean fluorescence intensity (MFI) plus two standard deviations (SDs) of the negative samples.

### Enzyme-linked immunosorbent assay (ELISA)

To detect mouse immune serum titers, PvETRAMP11.2 and PvEXP1 (2.5–5.0 μg/ml/sample) were coated on 96-well plates as previous description [21]. Briefly, after blocking with 5% nonfat dry milk in PBS-T, a two-fold serial dilutions of anti-PvETRAMP and anti-PvEXP1 mouse sera were add to each well, respectively. HRP-conjugated anti-mouse IgG antibodies (H+L) (dilution 1:10,000) (Pierce Biotechnology, Rockford, IL, USA) was added to each well as a secondary antibody for 1 h at 37°C. The reaction was developed by addition of 100 μl of diluted TMB solution (Invitrogen) for 15 min at 37°C, stopped with 100 μl 1 N HCl, and measured optical density (OD) at 450 nm. All samples were tested in duplicate, and the mean absorbance was calculated. The ELISA titer was the dilution at which the absorbance unit was nearest to 1.0.

### Indirect immunofluorescence assay (IFA)

*P*. *vivax* parasites were collected from malaria patients in Thailand and spotted onto 8-well glass slides. The slides were fixed with ice-cold acetone for 10 min, dried, and stored at -80°C until use. Before use, the slides were thawed on silica gel blue (Samchun Chemical, Pyeongtaek, Korea) and blocked with PBS-T containing 5% nonfat milk at 37°C for 30 min. As described elsewhere [22], slides incubated (single or double-labeled) with rabbit anti-PvMSP1-19 (1:200 dilution), rabbit anti-PvDBP (1:100 dilution), rabbit anti-Pv12 (1:200 dilution), rabbit anti-RhopH2 (1:200 dilution), mouse anti-PvETRAMP11.2 (1:100 dilution), rabbit anti-PvETRAMP11.2 (1:200 dilution), mouse anti-PvEXP1 (1:100 dilution), or rabbit anti-PvEXP1 (1:200 dilution) as primary antibodies at 37°C for 1 h. After the reaction of primary antibodies, the slides were stained with Alexa Fluor 546-conjugated goat anti-rabbit IgG or Alexa Fluor 488-conjugated goat anti-mouse IgG as secondary antibodies (Invitrogen), and nuclei stained with 4’,6-diamidino-2-phenylindole (DAPI, Invitrogen) at 37°C for 30 min. The slides were mounted in ProLong Gold antifade reagent (Invitrogen) and visualized under oil immersion in a confocal laser scanning FV200 microscope (Olympus, Tokyo, Japan) equipped with ×20 dry and ×60 oil objectives. Images were captured with FV10-ASW 3.0 viewer software and prepared for publication with Adobe Photoshop CS5 (Adobe Systems, San Jose, CA, USA).

### *In situ* proximity ligation assay (PLA)

We analyzed specific interaction between PvETRAMP and PvEXP1 proteins in *P*. *vivax* parasites and detected by *in situ* PLA assay using the Duolink In Situ kit (Olink Bioscience, Uppsala, Sweden). Slides smeared with parasite-infected blood samples were fixed with ice-cold acetone using the same method as for the previous IFA slides. These slides blocked with PBS-T containing 5% nonfat milk at 37°C for 60 min. For primary antibody reactions, the slides were double-labeled at 37°C for 1 h with the following antibodies: rabbit anti-PvETRAMP11.2 (1:200 dilution) and mouse anti-PvEXP1 (1:100 dilution), rabbit anti-PvMSP1 (1:200 dilution) and mouse anti-PvETRAMP11.2 (1:100 dilution), rabbit anti-PvRALP1 (1:200 dilution) and mouse anti-PvRON2 (1:100 dilution). The primary antibody solution was taped off from the slides, which were then washed in PBS-T. The slides were incubated with anti-mouse MINUS, anti-rabbit PLUS or PLA probes mixture in a humidity chamber for 1 h at 37°C. After washing, the slides were incubated with ligation mixture in a humidity chamber for 30 min at 37°C. The slide washed as above and incubated with amplification-polymerase solution in humidity chamber for 1.5 h at 37°C. After washing, the slides were mounted with a cover slip using a minimal volume of mounting medium with DAPI. All images were visualized and analyzed as above.

### Statistical analysis

Data were analyzed using GraphPad Prism (GraphPad Software, San Diego, CA, USA), SigmaPlot (Systat Software Inc., San Jose, CA, USA) and Microsoft Excel 2007 (Microsoft Corp., Redmond, WA, USA). Mann-Whitney *U*-tests were used to compare the differences between the means of each group for statistical significance. Statistical differences of *p* < 0.05 were considered significant. Simple scatter-regression was used to make a standard curve.

## Results

### Schematic primary structure of PvETRAMP11.2 and PvEXP1

*Pvetramp11*.*2 and pvexp1* gene sequence information encoded by PVX_003565 and PVX_091700 were located on chromosome 4 and 9, respectively. The sequences of two proteins had retrieved from PlasmoDB revealed that PvETRAMP11.2 (Fig 1A) and PvEXP1 (Fig 1B) were consisted of 110 and 148 AAs with predicted molecular masses of 12 and 15 kDa, respectively. PvETRAMP11.2 was encoded by a single exon gene. All two comprised an SP (AAs 1–22), and two TM domains were found in PvETRAMP11.2 (AAs 7–29 and 52–74) and PvEXP1 (AAs 4–22 and 77–99). To express these proteins successfully, all two target genes were cloned without the SP (Fig 1).

### Recombinant protein expression, purification and Western blot analysis of truncated PvETRAMP11.2 and PvEXP1

The recombinant proteins encoding truncated PvETRAMP11.2 and PvEXP1 (ΔSP) were successfully expressed, and purified under non-denaturing conditions as shown in schematic structure of Fig 1. The integrity and purity of the purified recombinant proteins were assessed by SDS-PAGE. Purified PvETRAMP11.2 and PvEXP1 migrated as single bands of ~16 kDa (Fig 1C) and ~22 kDa (Fig 1D) under reducing conditions.

**Fig 2:**
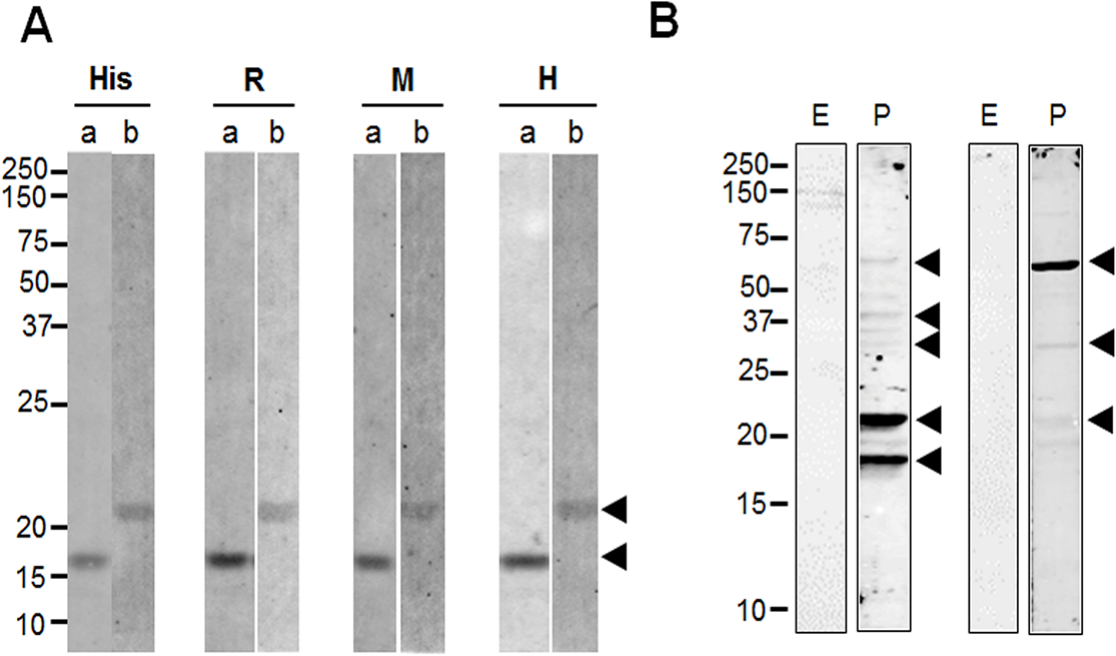
Western blot of recombinant PvETRAMP11.2 and PvEXP1 proteins, and *P*. *vivax* schizont extracts with specific antibodies. *A*. Western blot analysis recombinant proteins using anti-His antibody (His), rabbit immune sera (R), mouse immune sera (M), and vivax-infected human sera (H). a, PvETRAMP11.2; b, PvEXP1. Arrowheads indicate target bands specific to each recombinant protein. *B*. Anti-PvETRAMP11.2 (lane 2) and anti-PvEXP1 (lane 4), antibodies reacted with *P*. *vivax* schizont extracts, however, were not reacted with uninfected erythrocytes (lanes 1 and 3). Arrowheads indicate specific bands to each antibody.

The corresponding Western blot analysis revealed similar and specific patterns of migration for each antigen (Fig 2A). However, pre-immune mouse and rabbit sera samples used as negative controls did not reacted with the two recombinant protein antigens (data not shown). The specific single ~16 kDa band of PvETRAMP11.2 (Fig 2A, lane a) and ~22 kDa band of PvEXP1 (Fig 2A, lane b) were recognized by individual specific antibodies, suggesting that these antibodies were specific against two recombinant proteins, respectively.

Anti-PvETRAMP11.2 antibody recognized as ~18, ~22, ~32, 37 and ~67 kDa (Fig 2B, lane 2) and strongly recognized ~18 and ~22 kDa to parasite antigens. Anti-PvEXP1 antibody recognized a ladder of bands as ~22, ~32 and ~67 kDa (Fig 2B, lane 4) and strongly recognized the ~67 kDa band while weakly but little bit larger than predicted PvEXP1 (~22 kDa) protein in *P*. *vivax* parasites. However, uninfected erythrocyte antigens used as negative controls did not reacted with the two immune sera samples (Fig 2B, lanes 1 and *3*). These data suggest that PvETRAMP11.2 and PvEXP1 may form complexes with other proteins.

### Humoral immune response analysis of recombinant PvETRAMP11.2 and PvEXP1 proteins

**Fig 3:**
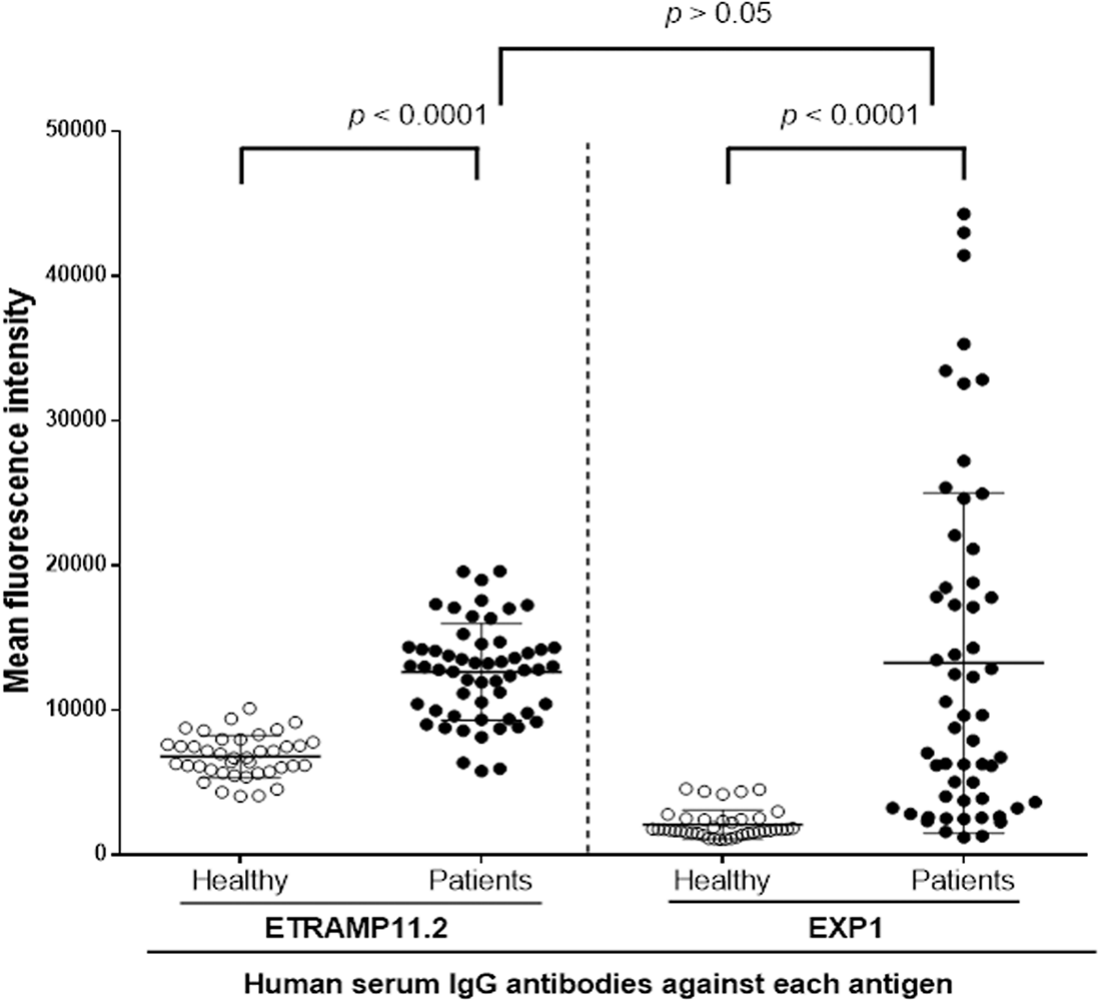
Total IgG against recombinant PvETRAMP11.2 and PvEXP1 proteins using protein microarrays. Immunoreactivity against each antigen with the sera of malaria patients (Patients) and healthy individual samples (Healthy) from Korea was determined. Each antigen was probed with the sera of 56 malaria patients and 40 healthy individuals. The *P* values were calculated using Mann-Whitney *U*-tests. The bar indicates the mean ± standard deviation. There were significant differences in the total prevalence of anti-PvETRAMP11.2, and anti-PvEXP1 IgGs between vivax patients and healthy individuals (*p* < 0.0001).

To evaluate humoral immune responses against PvETRAMP11.2 and PvEXP1 further, a protein array was used to screen for the presence of antibodies against purified proteins in vivax malaria patient sera. The responses of IgG antibodies against PvETRAMP11.2 and PvEXP1 in serum samples from 56 patients infected with *P*. *vivax* and 40 healthy individuals were determined. The prevalence of anti-PvETRAMP11.2 and PvEXP1 antibodies showed that the sensitivity was 76.8% and 69.6%, respectively, and the specificity was 97.5% and 87.5%, respectively (Table 1). These sera from *P*. *vivax-*exposed individuals showed a significantly higher MFI than from malaria naïve subjects (Fig 3, *p* < 0.0001).

**Table 1:** Prevalence (% positive), 95% confidence intervals, and mean fluorescence of intensity of IgG responses to *Plasmodium vivax* ETRAMP11.2 and EXP1 in vivax patients and healthy individual serum samples.

Protein	No. of patients samples (*n*)			95% CI^b^	MFI^c^	No. of healthy samples (*n*)			95% CI	MFI	*p*-value^e^
	Positive	Negative	Total (%)^a^	(%)		Positive	Negative	Total (%)^d^	(%)		
ETRAMP11.2	43	13	56 (76.8)	64.2–85.9	12,647	1	39	40 (2.5)	87.1–99.6	6,811	*p*<0.0001
EXP1	39	17	56 (69.6)	56.7–80.1	13,273	5	35	40 (12.5)	73.9–94.5	2,112	*p*<0.0001

^a^ Sensitivity: % positive in patient samples.

^b^ Confidence intervals.

^c^ MFI: mean fluorescence intensity.

^d^ Specificity: % negative control healthy samples.

^e^ Differences in the total IgG prevalence for each antigen between vivax patients and healthy individuals were calculated with Mann-Whitney *U*-test. *P* < 0.05 considered significant.

### IgG titers of mice immunized recombinant PvETRAMP11.2 and PvEXP1 proteins

**Fig 4:**
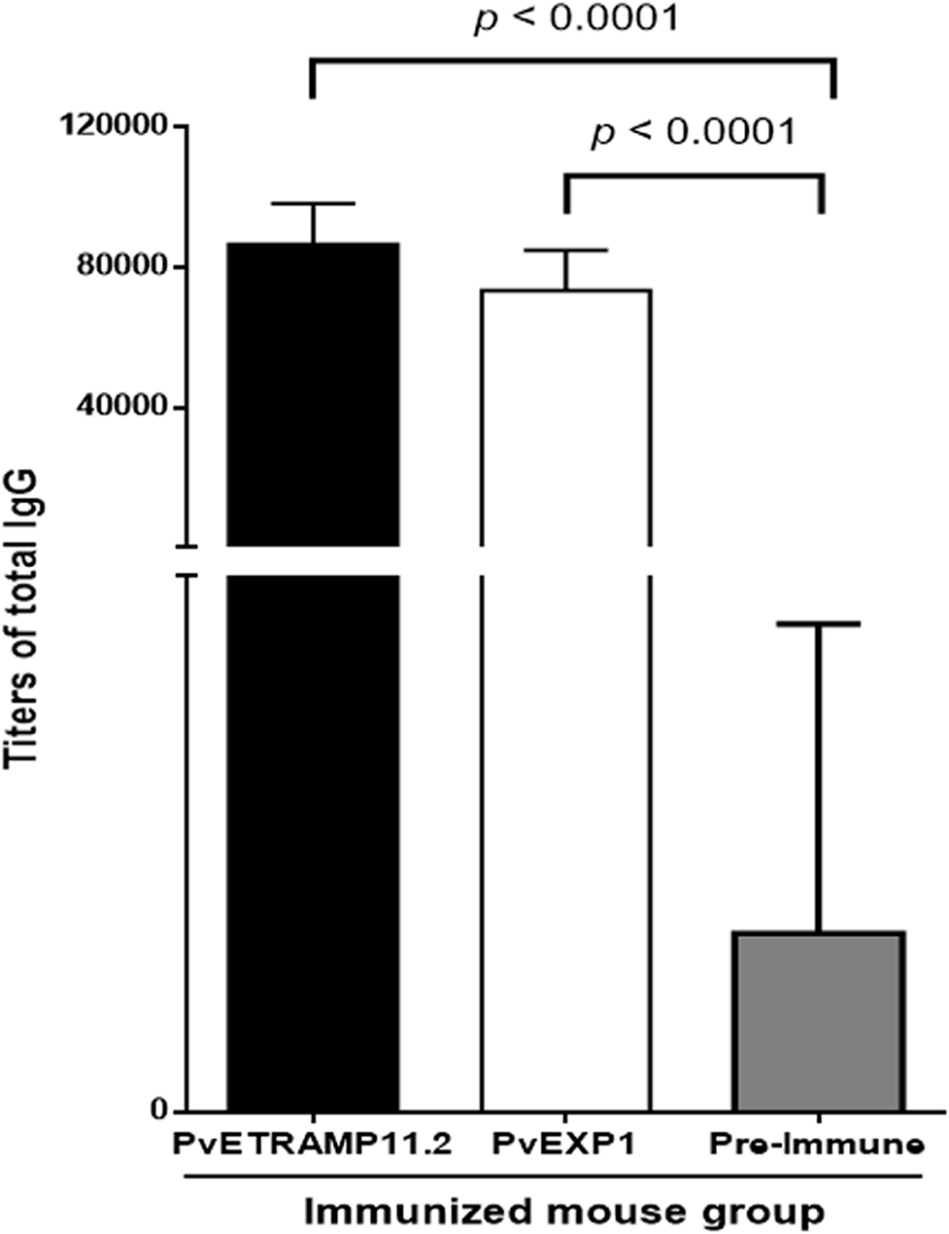
IgG responses against recombinant PvETRAMP11.2 and PvEXP1 proteins in immunized mice. Groups of three PvETRAMP11.2 (Black bar) and PvEXP1 (White bar) mice were immunized with the respective protein constructs, and titers of total IgG were evaluated by ELISA after three immunizations of PvETRAMP11.2 or PvEXP1, respectively. The total IgG titers against PvETRAMP11.2, and PvEXP1 proteins were significantly higher than those of pre-immune mice (*p* < 0.0001). Results are expressed as means ± SD.

After three immunizations with recombinant PvETRAMP11.2 or PvEXP1 proteins, the specific IgG titers of immune BALB/c mouse sera were determined by ELISA. The titers against PvETRAMP11.2 and PvEXP1 were 86,000 ± 12,000 and 73,000 ± 12,000 (mean ± SD), respectively (Fig 4) and were significantly higher than those of pre-immune mouse sera (*p* < 0.0001). Together with the strong immunoreactivity of vivax patient sera to each protein, these data strongly suggest that PvETRAMP11.2 and PvEXP1 induced a long-term potent immune response.

### Subcellular localization of PvETRAMP11.2 and PvEXP1

**Fig 5:**
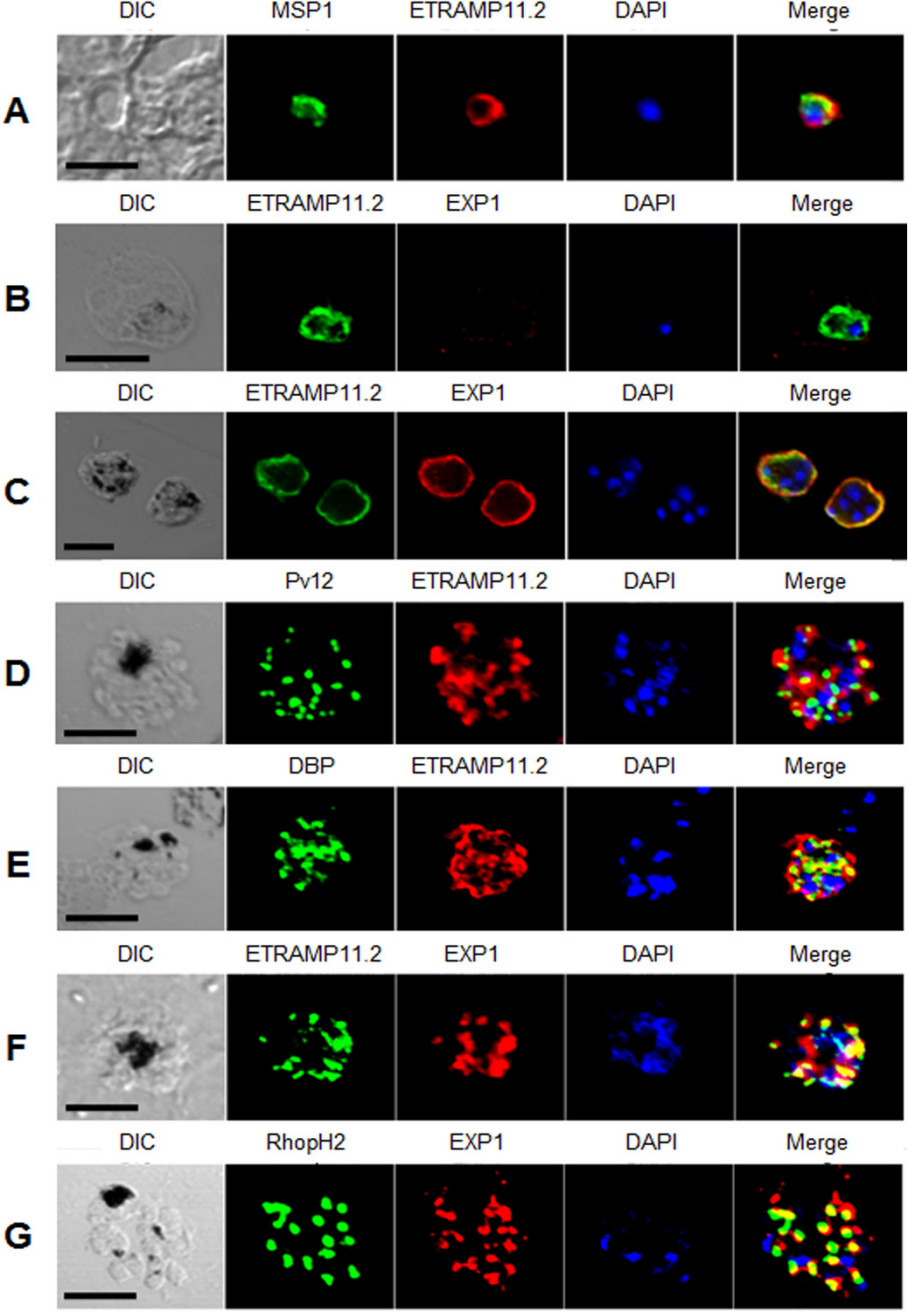
Subcellular localization of PvETRAMP11.2 and PvEXP1 proteins in *P*. *vivax* blood-stage parasites. The parasites were labeled with antisera against immune serum samples: PvETRAMP11.2 and PvMSP1 in ring stage (*A*), double-labeled with PvETRAMP11.2 and PvEXP1 in ring stage (*B*) and immature schizont stage (*C*). Parasites were double-labeled with antisera against PvETRAMP11.2 and Pv12 (*D*), PvETRAMP11.2 and PvDBP (*E*) in mature schizont stage, PvETRAMP11.2 and PvEXP1 (*F*) and PvEXP1 and PvRhopH2 (*G*), in mature schizont stage. Nuclei were visualized with DAPI in merged images. Scale bar represents 5 μm.

To determine the localization of PvETRAMP11.2, anti-PvETRAMP11.2 and anti-PvMSP1 antibodies were used for immunofluorescence analyses. Results showed that the PvMSP1 signal was surrounded by the PvETRAMP11.2 signal, but was within the iRBC (Fig 5A). However, the expression level of PvEXP1 was too low to detect in ring stage (Fig 5B). This indicated that PvETRAMP11.2 might be located in the intracellular parasite membrane in the ring stage. In addition, PvETRAMP11.2 partially colocalized on intracellular parasite membrane in the early schizont stage with PvEXP1 (Fig 5C). These results suggested that PvETRAMP11.2 and PvEXP1 were expressed on the apical organelles of merozoites in schizont-stage parasites, where they were released, and incorporated into the parasite membrane during invasion into erythrocytes and development until mature schizont stage. In mature schizont stage parasites, PvETRAMP11.2 and PvEXP1 were highly expressed on apical organelles in the schizont-stage parasites, however, PvEXP1 was not observed in the ring stage (Fig 5B). Immunofluorescence analyses of the mature schizont stage were performed using rhoptry-resident Pv12 and RhopH2 proteins [23], and microneme-resident DBP protein [24], which are well-known apical organelle markers. Most of PvETRAMP11.2 (Fig 5D–5G) and a part of PvEXP1 (Fig 5F and 5G) were found to localize in an apical pattern within merozoites. However, they were appeared to be different localizations from rhoptry and microneme organelles although they partially overlapped with Pv12, PvRhopH2 and PvDBP.

### Interactions between PvETRAMP11.2 and PvEXP1

**Fig 6:**
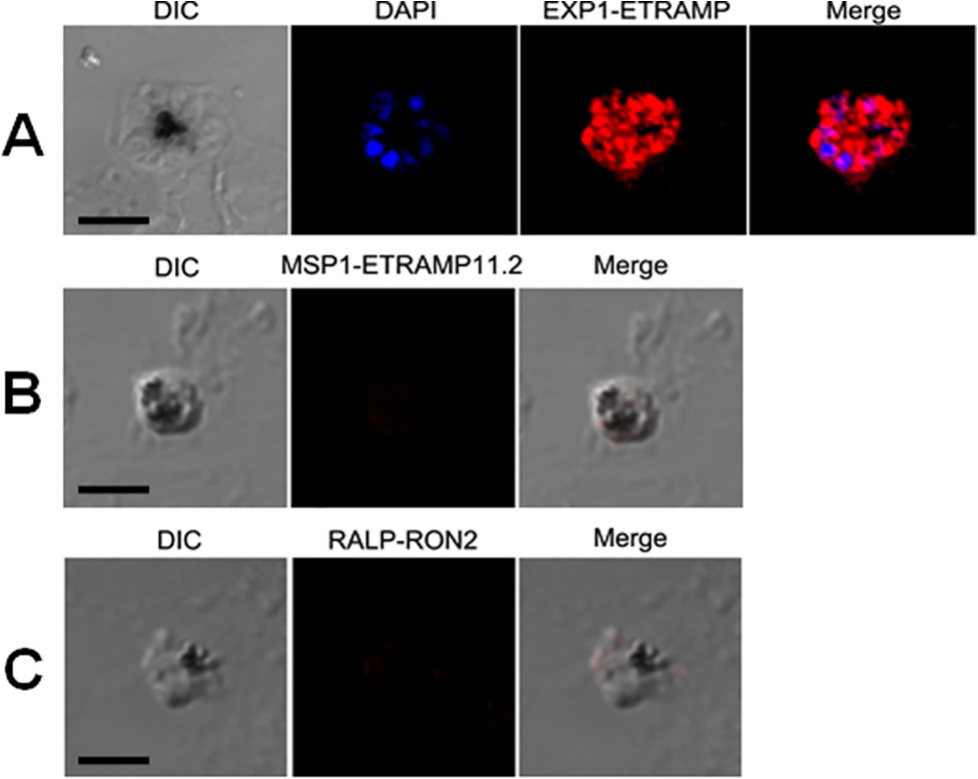
Protein-protein interactions between PvETRAMP11.2 and PvEXP1 were analyzed by *in situ* proximity ligation assay (PLA). PvETRAMP11.2 and PvEXP1 interactions were visualized by probing mouse and rabbit immune sera, and staining parasites with probes termed anti-mouse MINUS and anti-rabbit PLUS (*A*). Hybridization probes were labeled with Texas Red (Red), and nuclei were stained with DAPI (blue). Analysis of different localized proteins, PvETRAMP11.2 and PvMSP1 (*B*), and the rhoptry organelle localized proteins, PvRALP1 and PvRON2 (*C*) by PLA assay. Scale bar represents 5 μm.

To investigate possible interactions between PvETRAMP11.2 and PvEXP1 in native *P*. *vivax* parasites *in vivo*, we applied the PLA assay using mouse or rabbit immune sera as primary antibodies. We observed strong fluorescence signals in *P*. *vivax* parasites (Fig 6A), which suggested that PvETRAMP11.2 might interacted with PvEXP1 in immature to mature schizont stages. Comparatively, we could not found signal intensity between the merozoite surface-resident protein PvMSP1 [22] and PvETRAMP11.2 (Fig 6B) used as negative control. This might be because of their different localizations or because there was no interaction with each other. Additionally, we could not observe any specific interaction between PvRON2 [25] and PvRALP1 (Fig 6C), although both localized in rhoptry organelles of merozoites (data not shown). Conclusively, the signals detected in Fig 6 were specific, suggesting that PvETRAMP11.2 might interact with PvEXP1 in *P*. *vivax* parasites.

## Discussion

*Plasmodium* PVMs are established outside of the parasite and within the iRBCs. This serves as a barrier between the parasite and the iRBCs, which is critical for parasite survival and development within RBCs. At the molecular level, the PVM undergoes the following steps during parasite invasion into RBCs: (i) a coordinated cascade of signaling events [26] that leads to proteolytic processing of essential invasion-related molecules [27], (ii) the activation of motor proteins [28], (iii) the secretory release of proteins and lipids from the dense granules and rhoptry organelles [11, 29, 30], (iv) the localized alteration of erythrocyte membrane architecture with general membrane remodeling, (v) closure and “sealing” of the erythrocytes and PVMs, and (vi) nutrient acquisition [12]. Although PVM is enigmatic, the ETRAMPs constitute the largest family of few known PVM proteins, and are frequently expressed at high levels [4]. Previously, PfEXP1 was shown inserted into the PVM with its C-terminus facing the host cell cytoplasm [13]. To date, however, PVM information in *P*. *vivax* is unknown. Here, we first characterized two immunogenic proteins homologue to PVM-associated proteins in *P*. *falciparum*, termed PvETRAMP11.2 (homologue of PfETRAMP2/PfETRAMP11.2) and PvEXP1.

PfETRAMP2, together with other PfETRAMPs, was previously found to form membrane-dependent homo-oligomeric complexes at the PVM that are distinct from EXP1 [14]. One of *P*. *yoelii* ETRAMP (PyETRAMP) was found in apical organelles in late schizont-stage parasites [31]. However, there is no evidence to indicate that PfETRAMPs localize at apical organelles in blood-stage parasites. In the present study, PvETRAMP11.2 was detected at apical organelles in the merozoites, where partially colocalized with PvEXP1 (Fig 5). Moreover, PvETRAMP11.2 appeared to localize at the intracellular parasite membrane in ring and immature schizont stage, where it still colocalized with PvEXP1. PvEXP1 could not be detected in the ring stage, indicating the expression profile of the two proteins might be different in blood-stage parasites. In comparisons with intracellular localization of known apical organelle proteins, PvETRAMP11.2 and PvEXP1 colocalized with either rhoptry or microneme proteins in mature schizont-stage parasites. As such, we proposed that PvETRAMP11.2 and PvEXP1 colocalized at the intracellular parasite membrane or perhaps in PVM in different blood-stage parasites.

Interestingly, PvETRAMP11.2 and PvEXP1 possessed a ladder of bands (Fig 2B). Previously, PfETRAMPs and PfEXP1 were shown that they formed complexes in live parasites [14], and these findings were also confirmed *P*. *berghei* ETRAMPs [32, 33]. Thus, our results suggest that PvETRAMP11.2 and PvEXP1 also might form the complex-like structure. Additionally, PfETRAMPs also produced polymer and/or complex by themselves [14], hence, the other bands recognized in PvETRAMP11.2 properties possibly caused by PvETRAMPs complex or polymer. To further analyze the possible interaction *in vivo* among PvETRAMP11.2 and PvEXP1, we developed PLA assay as previous report [34] (Fig 6). Strong fluorescence signals were detected in each two proteins, suggesting that PvETRAMP11.2 might interact with PvEXP1. These results also confirmed the possible complex found in Western blot analysis between PvETRAMP11.2 and PvEXP1.

In Western blot analysis of native parasite antigen with anti-PvETRAMP and anti-PvEXP1 antibodies, a ~67 kDa band was detected by both anti-PvETRAMP11.2 and anti-PvEXP1 antisera, which seemed that both PvETRAMP11.2 and PvEXP1 were part of a protein complex present in native parasite. In addition, two specific bands about 18 and 22 kDa were strongly recognized with anti-PvETRAMP antibody and weakly with anti-EXP1 antibody. Although similar size of these bands are unknown without immunoprecipitation study, it is theoretically possible that there is complex formation between PvETRAMP11.2 and PvEXP1 but practically is difficult to confirm those of experiments because of difficulty of vivax parasite culture.

*P*. *vivax* possesses nine members in the *etramp* family. All of these genes have putative orthologs in *P*. *falciparum*, and *pvetramps* members have an SP, TM domain, and polymorphic C-terminal domains [31]. Previously, PvETRAMP11.2 was identified by its immunogenic profile from *P*. *vivax* blood-stage candidates [15]. This ortholog was identified as PfETRAMP2/PfETRAMP11.2 (PlasmoDB, PF02_0025/PF11_0040) in *P*. *falciparum*. Recently, PfETRAMP11.2 was described as immunological reactive protein, which was suggested as an immunologically reactive protein and was suggested to be a potential vaccine candidate for blood-stage malaria [35]. In the present study, wheat germ cell-free expression system and protein array were used to express recombinant proteins and characterize antibody reactivity of *P*. *vivax* infection. The antigenicity and immunogenicity of PvETRAMP11.2 and PvEXP1 proteins were evaluated by the large number of patients sera samples (Figs 3 and 4, Table 1), comparatively, the antigenicity of each protein confirmed the previous identification of immunoprofiles of PvETRAMP11.2 [15] and PvEXP1 [15].

In conclusion, we have elucidated the expression of PvETRAMP11.2 and PvEXP1 on the parasite membrane of parasite-infected RBCs and their potential molecular interaction in *P*. *vivax* blood-stage parasites. We used a protein array to evaluate the antibody responses against these two *P*. *vivax* proteins in vivax malaria patients. The colocalizations and interactions of PvETRAMP11.2 and PvEXP1 were first proven *in situ* in *P*. *vivax* blood-stage parasites. These observations will greatly advance our understanding of possible protein-protein complex formations in vivax malaria parasites, and it need to be required further work.
